# Towards a Miniaturized Culture Screening for Cellulolytic Fungi and Their Agricultural Lignocellulosic Degradation

**DOI:** 10.4014/jmb.2007.07005

**Published:** 2020-08-27

**Authors:** Jantima Arnthong, Chatuphon Siamphan, Charuwan Chuaseeharonnachai, Nattawut Boonyuen, Surisa Suwannarangsee

**Affiliations:** National Center for Genetic Engineering and Biotechnology (BIOTEC), National Science and Technology Development Agency (NSTDA), 113 Thailand Science Park, Klong Luang, Pathumthani 12120, Thailand

**Keywords:** Lignocellulosic biomass, microplate-based screening, enzymatic saccharification, cellulase, hemicellulase, fungal degradation

## Abstract

The substantial use of fungal enzymes to degrade lignocellulosic plant biomass has widely been attributed to the extensive requirement of powerful enzyme-producing fungal strains. In this study, a two-step screening procedure for finding cellulolytic fungi, involving a miniaturized culture method with shake-flask fermentation, was proposed and demonstrated. We isolated 297 fungal strains from several cellulose-containing samples found in two different locations in Thailand. By using this screening strategy, we then selected 9 fungal strains based on their potential for cellulase production. Through sequence-based identification of these fungal isolates, 4 species in 4 genera were identified: *Aspergillus terreus* (3 strains: AG466, AG438 and AG499), *Penicillium oxalicum* (4 strains: AG452, AG496, AG498 and AG559), *Talaromyces siamensis* (1 strain: AG548) and *Trichoderma afroharzianum* (1 strain: AG500). After examining their lignocellulose degradation capacity, our data showed that *P. oxalicum* AG452 exhibited the highest glucose yield after saccharification of pretreated sugarcane trash, cassava pulp and coffee silverskin. In addition, *Ta. siamensis* AG548 produced the highest glucose yield after hydrolysis of pretreated sugarcane bagasse. Our study demonstrated that the proposed two-step screening strategy can be further applied for discovering potential cellulolytic fungi isolated from various environmental samples. Meanwhile, the fungal strains isolated in this study will prove useful in the bioconversion of agricultural lignocellulosic residues into valuable biotechnological products.

## Introduction

The significant global concern regarding carbon dioxide (CO_2_) emissions from the combustion of fossil fuels into the atmosphere has promoted the use of biofuel-derived biomass feedstock as an eco-friendly, alternative energy source [1, 2]. The utilization of waste biomass is not only a carbon neutral process but such resources can also be considered inexpensive, abundant and sustainable [3]. Lignocellulosic biomasses, *i.e.*, sugarcane bagasse and trash and wheat straw and rice straw, can be used to produce a wide range of biobased chemicals used in biofuels and construction [4, 5]. The integration of lignocellulosic residues into the circular economy can scale up sustainable bioproducts, bioenergy development and biotechnological interest [6-8].

Lignocellulosic feedstocks mainly comprise cellulose, hemicellulose, and lignin, which form their rigid structure [9]. The composition of lignocellulose is diverse depending on the plant species, growth condition and age [10]. Basically, physicochemical pretreatment, recalcitrant structure disruption and enzymatic saccharification steps are required for the transformation of lignocellulosic biomass into fermentable sugars, which can then be further converted to biofuel or other value-added chemicals [11]. Cellulases are a group of enzymes that catalyze cellulose as a major component of lignocellulose in which it breaks the 1,4-β-glycosidic bonds within the cellulose chain of lignocellulosic biomass into glucose subunits [12]. Cellulases consist of three enzymes: endoglucanase (EG; E.C. 3.2.1.4), cellobiohydrolase (CBH; E.C. 3.2.1.91), and β-glucosidase (BGL; E.C. 3.2.1.21), which act synergistically in cellulose hydrolysis [13]. Additionally, the action of endoxylanase (E.C.3.2.1.8) and β-xylosidase (E.C.3.2.1.37) is essential for the hydrolysis of xylan as the second most abundant polysaccharide in lignocellulosic materials [14]. Cooperative digestion of cellulases and hemicellulases is required for efficient hydrolysis of lignocellulose [15, 16]. Even though the preparation of commercial cellulase supplemented with hemicellulase is available on the market, significant research efforts are still focused on finding a novel cellulase cocktail [17, 18]. This might be due to the cost of enzymes and the heterogeneity of lignocellulosic substrates, resulting in single-enzyme cocktails being unsuitable for all applications [19]. Finding an ideal microbial strain that produces an efficient enzyme cocktail capable of reducing the cost of enzymes and consequently the cost of biomass saccharification is still a challenging task [20].

Cellulases can be produced by several types of microorganisms, including bacteria and fungi [21]. For example, fungal genera, *i.e.*, *Trichoderma*, *Penicillium*, *Aspergillus* and *Talaromyces*, have been proven to be better candidates for cellulase production than other microbial species since these fungi can secrete large amounts of cellulases and hemicellulases [22]. The conventional technique for primarily screening potential high cellulase producers is the agar-plate method, in which cellulosic material is added into an agar plate as a selective carbon source [23]. However, the context of screening from a large number of fungal strains using the agar-plate screening technique is limited, laborious and time consuming. Recently, different high-throughput screening platforms for filamentous fungi have been developed, ranging from the utilization of robotic machines to droplet-based microfluidic technology [24, 25]; nevertheless, these expensive instruments cannot possibly be installed in all laboratories.

**Fig. 1:**
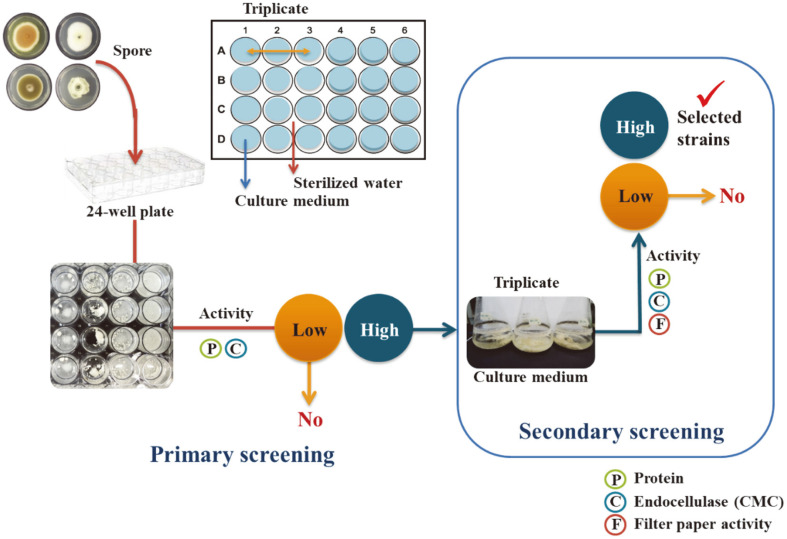
Schematic of the screening workflow for cellulolytic fungi. Two proposed steps: (1) preliminary screening using a microplate-based cultivation step; (2) secondary screening using shake-flask cultivation.

A miniaturized culture method that scales down the fermentation volume into a 24-well microtiter plate has been reported to efficiently reduce labor and facilitate experiments with a large number of fungal strains [26]. This method was successfully demonstrated in solid cultivation of *Trichoderma* strains for screening of cellulase activity [11], screening of *A. carbonarius* for citric acid production [26] and screening of *A. terreus* mutants for itaconic acid production [27]. In this study, we applied the 24-well plate culture technique to discover natural fungal strains with cellulose degradation capabilities, as summarized in Fig. 1. Cellulolytic fungi were isolated from various lignocellulosic samples collected from two selected different areas in Thailand. For the first screening step, these fungi were primarily assessed for their cellulase activities by a microplate-based culture method that mimics shake-flask conditions. For the second step, selected fungal strains were subsequently screened by shake-flask fermentation. Additionally, potential cellulase-producing strains were identified using DNA sequencing of the nrDNA internal transcribed spacer (ITS) region. To support ITS data and for use as additional molecular fungal markers, loci of *CaM*, *TEF1α*, *RPB2* and other regions were also employed for further identification. Finally, their secreted enzymes based on the hydrolysis of different agro-industrial residues into simple fermentable sugars were evaluated.

## Materials and Methods

### Sampling and Isolation of Cellulolytic Fungi

Twenty-two samples of various cellulose-containing substrates, including rice husks, rice husk ash, dried butterfly peas, dried banana stems, organic fertilizer, plant debris, manure, soil, and vermicompost, were randomly collected from two selected locations in Thailand: 1) Rural Farming Happiness Center, Mueang Ratchaburi district, Ratchaburi Province (13°27'N, 99°45'E) and 2) Tha Makham Natural Farming Center, Mueang Kanchanaburi district, Kanchanaburi Province (14°31'N, 99°29'E). All samples were transferred to the laboratory at BIOTEC and maintained at 4°C in a refrigerator.

Modified Czapek-Dox’s agar medium supplemented with 1% (w/v) carboxy-methyl cellulose (CMC) as the sole carbon source was applied to isolate cellulose-degrading fungi. This modified medium contained the following (g/l): CMC, 10.0; NaNO_3_, 2.0; K_2_HPO_4_, 1.0; MgSO_4_, 0.5; KCl, 0.5; FeSO_4_, 0.01; and agar, 15.0 (pH 6.8). Rose bengal (Sigma-Aldrich, Spain) and 30 ppm streptomycin sulfate (BIO Basic, Markham, Canada) were added to the modified medium. Fungal strains were isolated on the modified media plates and incubated at room temperature (~25–28°C) until fungal appearance. Distinct fungal colonies with different morphological forms, *i.e.*, colony color, texture and margins, were subcultured and preserved on potato dextrose agar (PDA) plates for further studies.

### Primary Screening by a Microplate-Based Method

The miniaturized culture system was carried out in a 24-well plate (Nest Biotechnology, China). The diameter of each well was 1.9 cm^2^ with a working volume of 1 ml per well. Spores of each fungal isolate were prepared by a five-day cultivation on PDA plates at 30°C. Then, fungal spores were inoculated using a sterile disposable toothpick to transfer every strain into the 24-well plate containing 1 ml of the screening medium in each well. Mandels and Weber medium (MM; [28]), containing 1% Avicel as the sole carbon source, was applied as a screening medium. The 24-well culture plates were installed in plate stacks and then incubated at 30°C and 250 rpm for 7 days. An amount of sterilized distilled water was added into the free space between well plates to avoid evaporation during the incubation period. The fungal cultures were harvested by centrifugation at 3,000 ×*g* for 10 min at 4°C. The enzyme solutions were kept at 4°C until further analysis. As in a previous study, *A. aculeatus* BCC199 was used for comparison as a cellulase-producing reference strain [13].

### Secondary Screening through 250-ml Shake-Flask Fermentation

Based on the primary screening method, cellulase production of selected fungal strains was validated in 250-ml shake flask cultures. Each fungal strain was plated on PDA medium and incubated at 30°C for 5 days. Subsequently, spore suspensions were obtained by adding 0.1% (v/v) Tween 20 into the PDA plates. The fungal strains (1 × 10^8^ spores) were inoculated into 50 ml of MM medium containing 1% Avicel as the sole carbon source. The experiment was performed in triplicate and incubated at 30°C and 200 rpm for 7 days. The crude enzyme products were obtained by centrifugation of the culture at 9,000 ×*g* for 10 min at 4°C.

### Sequence-Based Identification of Cellulolytic Fungi

After the primary and secondary screenings, potent fungal strains were identified by molecular data. DNA extraction from a mycelium on PDA was performed using the method described by [29] with some modifications. The gene region of the ITS was sequenced first as a fungal barcoding locus. In case of unsuccessful identification and for supporting ITS data, unidentified fungal strains were further sequenced using one of the additional molecular loci, *i.e.*, translation elongation factor 1α (*TEF-1α*), the second largest subunit of RNA polymerase II (*RPB2*), calmodulin (*CaM*) or ß-tubulin (*TUB*). PCR products were amplified using the primer pairs ITS5/ITS4 [30], 983F/2218R [31], f*RPB2*-5F2/f*RPB2*-7cR [32], cmd5/cmd6 [33] and Bt2a/Bt2b [34]. All PCRs were performed in a Bio-Rad T100 Thermal Cycler (Bio-Rad, Hercules, CA, USA) according to conditions described by [35]. DNA sequencing was performed with the primers mentioned above at Macrogen Inc. (Korea). Sequences were analyzed by BioEdit v7.0.5.3 [36] and run through a standard nucleotide BLAST (Basic Local Alignment Search Tool; NCBI nucleotide database; http://www.ncbi.nlm.nih.gov/) to evaluate the similarity with reported sequences of fungal species. Fungal sequences were then analyzed by comparison with the type species sequences. All new fungal sequences were deposited in the NCBI database under the accession numbers shown in Table S1.

### Production of Cellulases in Submerged Fermentation

The selected fungal strains were cultivated under submerged fermentation in 250-ml shake flasks containing 50 ml of production medium. The production medium (1 L) was composed of the following: Avicel, 10 g; wheat bran, 25 g; KH_2_PO_4_, 2 g; CaCl_2_·2H_2_O, 0.3 g; urea 0.3 g; MgSO_4_·7H_2_O, 0.3 g; (NH_4_)2SO_4_, 1.4 g; FeSO_4_·7H_2_O, 0.005 g; MnSO_4_. H_2_O, 0.0016 g; ZnSO_4_·7H_2_O, 0.0014 g; CoCl_2_·2H_2_O, 0.002 g; peptone, 0.25 g; yeast extract, 0.1 g; and Tween, 80 0.1 g [37]. Each flask was inoculated with 5×10^6^ spores/ml spore suspension and incubated at 30°C under shaking at 200 rpm for 7 days. After cultivation, the solid part containing fungal mycelia and insoluble materials was removed by filtration through a nylon cloth, followed by centrifugation at 9,000 rpm and 4°C for 10 min. The crude enzyme solution was evaluated for enzyme activities and used for biomass saccharification experiments. All fungal cultivations were performed in triplicate.

### Enzymatic Saccharification of Agricultural Residues

All lignocellulosic materials, sugarcane trash (ST), sugarcane bagasse (BG), cassava pulp (CP) and coffee silver skin (SS), were locally obtained from selected sites located in Thailand. The biomass was physically processed using a cutting mill (Retsch SM2000, Germany) and sieved to particles 250-420 µm in size. Before hydrolysis, the lignocellulosic materials were pretreated by a two-stage integrated process [38]. In the first step, each agricultural residue was pretreated with diluted sulfuric acid (1% v/v) with a solid:liquid ratio of 1:2 at 121°C for 45 min to hydrolyze hemicellulose. For the second step, a 4% (w/v) sodium hydroxide solution was applied to degrade lignin at a solid:liquid ratio of 1:20, followed by heating at 121°C for 30 min. Then, the solid fraction was separated and washed with tap water until the pH was 7. The pretreated biomasses were dried at 70°C before enzymatic hydrolysis. Chemical compositions (percentage of cellulose, hemicelluloses and lignin) were analyzed using the standard NREL method [39].

For the enzymatic hydrolysis experiment, 5% (w/v) of pretreated biomass (ST, BG, CP or SS) was subjected to a 10-ml reaction consisting of 7.5 mg protein/g biomass crude enzyme, 50 mM sodium acetate buffer at pH 5.0, and 0.1% (v/v) sodium azide. The reaction was incubated at 50°C with shaking at 200 rpm for 72 h. The hydrolysate was centrifuged at 9,000 rpm for 10 min, and the supernatant was collected to analyze the released reducing sugar concentration using the 3,5–dinitrosalicylic acid (DNS) method [40]. The fermentable sugar profiles were analyzed via high-performance liquid chromatography (HPLC). The experiments were performed in quadruplicate.

### Determination of Enzyme Activity

The activity of total cellulases (FPase) was determined based on the International Union of Pure and Applied Chemistry (IUPAC) method using Whatman No. 1 filter paper as a substrate [41]. The reaction mixture was incubated at 50°C for 60 min. For CMCase and xylanase activities, enzymatic assays were conducted in a reaction mixture comprising 50 mM sodium acetate buffer (pH 5.0) using 1% (w/v) CMC and 1% (w/v) xylan from beech wood as the substrate. The amount of reducing sugar was quantified according to the DNS method [40]. One enzymatic activity unit (U) was defined as the amount of enzyme required to release 1 μmol of glucose or xylose per minute under the assay conditions. For determination of β-glucosidase (BGL) and β-xylosidase (BX) activity, 40 mM *p*-nitrophenyl-β-D-glucopyranoside (*p*NPG; Sigma) and *p*-nitrophenyl-β-D-xylopyranoside (pNPX; Sigma) were applied as the substrates, respectively. The assays were performed as previously described [13]. One BGL or BX activity U was defined as the amount of enzyme that liberated 1 μmol of p-nitrophenol per minute under the assay conditions. The total protein content was determined by using Bradford’s method with the Bio-Rad protein assay reagent (Bio-Rad, USA) and bovine serum albumin (BSA) as a standard protein. All experiments were performed in triplicate.

For the primary screening step, determination of cellulase activity (CMCase) was adapted by using a polypropylene 96-well microplate (Eppendorf, Germany). The small-scale assay was performed with a reaction mixture containing 10 μl of enzyme sample and 64 μl of 2% (w/v) CMC in 50 mM sodium acetate buffer (pH 5.0). After incubation at 50°C for 20 min, 126 μl of DNS solution was added to the reaction, and then the plate was placed in a water bath (90°C) for 10 min. Subsequently, the plate was placed on ice for 10 min. One hundred microliters were transferred into a new 96-well plate. The absorbance was measured on a microtiter plate reader at a wavelength of 540 nm.

## Results and Discussion

### Isolation and Preliminary Screening of Cellulase-Producing Fungal Strains

**Fig. 2:**
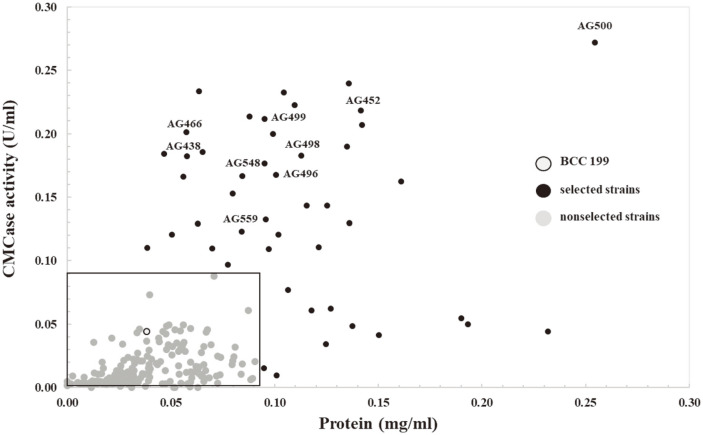
Scatter plot representing CMCase activity using a small-scale assay and the amount of secreted protein of different fungal strains based on a microplate-based screening assay. *A. aculeatus* BCC199 (reference strain; black border dot), selected strains (black dot), non-selected strains (gray dot) are shown.

A total of 297 fungal strains were isolated and cultivated on the 24-well plate in the small-scale assay. As shown in Fig. 2, most fungal isolates were able to secrete cellulases, and total protein contents were lower or comparable to that of *A. aculeatus* BCC199 as a reference strain (black border dot; [13]). To isolate and screen potential cellulase-producing strains, primary screening on solid agar plates might not guarantee enzyme production when compared with liquid-stage culture screening. Most industrial enzyme production has been performed under submerged cultivation [42]. Hence, it is essential to mimic large-scale experiments and simulate the culture conditions of submerged fermentation to discover potent fungal candidates. A 24-well microplate cultivation experiment was carried out to establish and screen a large number of fungal isolates in this study. With respect to finding the maximum cellulase producers, we selected 45 fungal strains (black dot) out of 297 strains because they presented higher cellulase activities and/or protein contents than those of *A. aculeatus* BCC199. These fungi accounted for 15% of the total number of fungal isolates.

### Secondary Screening of Cellulase-Producing Fungal Strains

**Fig. 3:**
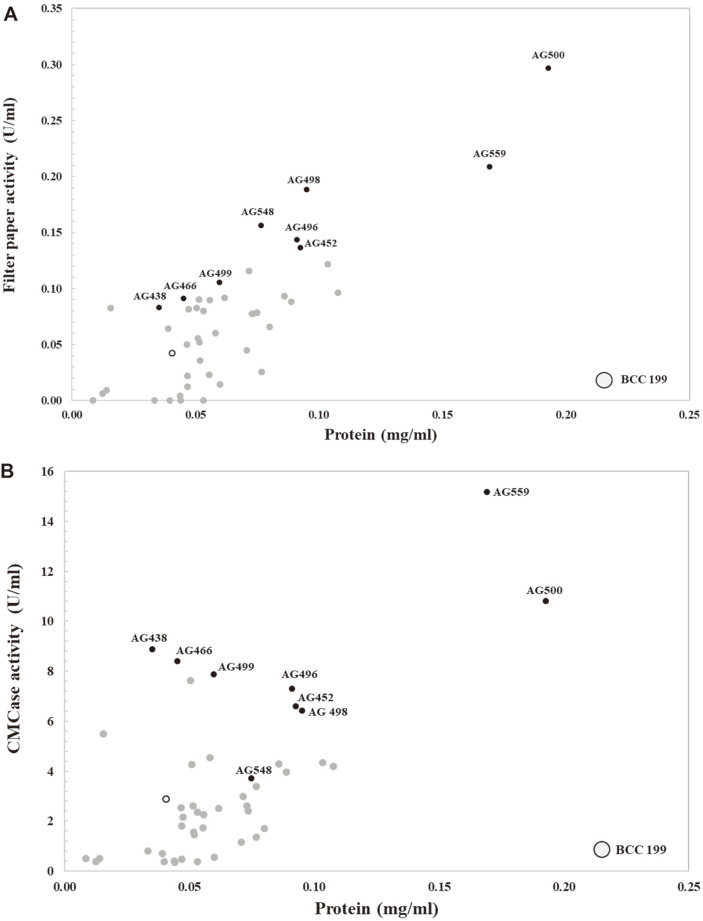
Scatter plot representing total protein content and cellulase activity against filter paper (FPase; A) and CMC (CMCase; B) as substrates of crude enzymes from selected fungal strains in a 250-ml shake flask. *A. aculeatus* BCC199 (reference strain; black border dot), selected strains (black dot), nonselected strains (gray dot) are shown.

To validate the preliminary screening step, 45 fungal isolates were inoculated in the same medium, but we scaled up the culture size to 50 ml in 250-ml shake flasks together with *A. aculeatus* BCC199 as a reference strain [13]. After cultivation, crude enzymes were prepared. Cellulase activities on CMC and filter paper substrate were assessed. As shown in Fig. 3, 18 and 31 strains produced higher CMCase and FPase activities, respectively, than those of *A. aculeatus* BCC199. Among them, strain AG500 exhibited outstanding cellulase activity and protein production after testing based on both the primary and secondary screening steps. This result indicated that the pattern of cellulase activity was in accordance with the preliminary screening result. Only 14 strains (31% of all strains) presented lower cellulase activities or lower protein secretion than those of *A. aculeatus* BCC199. These data may be due to the powerful microplate-based cultivation approach used in this study. This method may not only quantitatively determine the amount of certain enzyme activities but also eliminate fungal isolates that do not grow well under submerged fermentation conditions. For instance, the microplate-based cultivation method has been implemented successfully, as previously reported in other applications, such as for enzymatic hydrolysis of lignocellulosic biomass according to [43], for mutant archaea and fungal strains using mutagenesis [25, 44] and for fermentation production of citric acid, ethanol and glycerol by filamentous fungi [26].

Based on the secondary screening results, 9 strains out of 45 fungal strains were selected according to the high production level of cellulases (CMCase or FPase). The potential fungal strain numbers were AG438, AG452, AG466, AG496, AG498, AG499, AG500, AG548, and AG559. For CMCase activity, two fungal strains, AG559 and AG500, displayed the highest activities of 15.17 and 10.79 U/ml, respectively, while the reference strain (*A. aculeatus* BCC199) produced only 2.88 U/ml. Similarly, the highest FPase activities of 0.30 U/ml and 0.21 U/ml were produced by AG500 and AG559, respectively. Subsequently, these 9 fungal potential strains were identified using molecular identification and enzyme production. Fungal colonies of these fungal strains are shown in Fig. S1.

### Molecular Identification of Selected Cellulase-Producing Fungal Strains

As shown in Table 1, the BLAST search results of 9 cellulolytic enzyme-producing fungal strains were classified into 4 genera in 4 taxa as follows: *A. terreus* (AG466, AG438, and AG499), *P. oxalicum* (AG452, AG496, AG498, and AG559), *Ta. siamensis* (AG548) and *T. afroharzianum* (AG500). To our knowledge, this is the first report of cellulase production by *Ta. siamensis* and *T. afroharzianum* and on their ability to degrade lignocellulose. In addition, two well-known cellulase producers under the same genera, *Ta. cellulolyticus* and *T. reesei*, have been formerly reported [45, 46]. Furthermore, 3 out of these 9 potential strains, *A. terreus* strains, were found to be fungal risk group strains of biosafety level two (BSL2) based on the Thailand Biosafety Guidelines for Modern Biotechnology and Fungal Risk Assessment Reports (http://www.biotec.or.th/biosafety/;[47]). As a consequence, these strains were then excluded from this study to avoid the spreading of spores to indoor and outdoor environments. For subsequent enzyme production, only 6 fungal strains were chosen.

### Enzyme Production of Cellulase-Producing Fungal Strains

The application of cellulolytic microorganisms to cellulosic biomass bioconversion is considered one of the potential sustainable approaches to develop several bioproducts, such as biofuels [48]. The most common filamentous fungal genera for the production of cellulolytic enzymes were *Aspergillus*, *Trichoderma*, and *Penicillium* [49]. In this study, six fungal strains of three genera, *Penicillum*, *Trichoderma*, and *Talaromyces*, were grown under submerged fermentation in 250-ml shake flasks. In the experiment, Avicel and wheat bran were used as two coinducers for cellulolytic enzyme production. The presence of complex cellulose from plant materials such as wheat bran was demonstrated to be more efficient than pure cellulose in inducing the expression of lignocellulose-degrading enzymes [50]. Table 2 shows the enzyme activity profile of crude enzymes obtained after 7 days of cultivation. It was found that all fungal strains secreted higher cellulase activities when grown in a medium supplemented with wheat bran. Similarly, two fungal strains, *Penicillium* sp. CR-316 and *Penicillium* sp. CR-313, cultivated in medium containing rice straw as an inducer showed improved cellulase activities [51].

Of all strains tested, the *P. oxalicum* strains AG498 and AG496 exhibited the highest FPase activity of 0.56 and 0.53 IU/ml, respectively, whereas *A. aculeatus* BCC199 (the reference strain) produced an FPase activity of 0.24 IU/ml. This result was different from the secondary screening result, where AG500 and AG559 were superior in FPase and CMCase secretion. This might be because of the different compositions in the fungal medium. In addition, three strains of *P. oxalicum*, AG496, AG498, and AG452, produced higher levels of BGL activity than those of the other strains tested in this study. Different strains of *P. oxalicum* have been demonstrated to be excellent cellulase producers for biomass saccharification applications [17, 52]. Additionally, the highest activities of xylanase and β-xylosidase were also achieved with *P. oxalicum* (AG496 and AG498), contributing to the potential role of these strains in the production of biomass-degrading enzymes. *T. afroharzianum* AG500 showed notable FPase and CMCase activities in the secondary screening step; however, it exhibited quite low BGL activity when cultivated in the production medium. It is known that fungal strains of the genus *Trichoderma*, *i.e.*, *T. reesei*, produced BGL in small quantities compared to cellobiohydrolase and endoglucanase produced by the same species [46]. It should be noted that BGL activity plays a prominent role in the catalysis of cellobiose into glucose, overcoming cellobiose-mediated repression of cellulases [53]. This might be affected by the further hydrolysis capability of this fungal enzyme.

### Pretreatment of Lignocellulosic Substrates and Enzymatic Saccharification Using Crude Enzymes from Cellulolytic Fungi

In this study, various lignocellulosic agro-industrial byproducts, *i.e.*, sugarcane bagasse and trash, cassava pulp and coffee silverskin (a major waste product in the coffee-roasting industry) [54], were collected and used as raw materials for biomass saccharification. Lignocellulose has a complex molecular structure, which can block enzymes and prevent biomass degradation into fermentable sugars. The pretreatment step was performed to remove part of the lignin and hemicellulose and provide easier enzyme accessibility to cellulose [38, 55, 56]. All agro-industrial byproducts were subjected to the two-stage pretreatment process in which the biomass was first treated with diluted sulfuric acid followed by sodium hydroxide pretreatment. The chemical composition of untreated and pretreated lignocellulosic biomasses is illustrated in Table 3. It was clear that the two-stage pretreatment could increase the cellulose proportion and decrease the hemicellulose and lignin contents in lignocellulosic biomasses. The characteristics of lignocellulosic biomass before and after pretreatment are shown in Table S2. Previous studies suggested that the two-stage acid/alkali pretreatment efficiently improved the enzymatic digestibility of the pretreated biomass, resulting in a high conversion yield of fermentable glucose [57, 58].

**Fig. 4:**
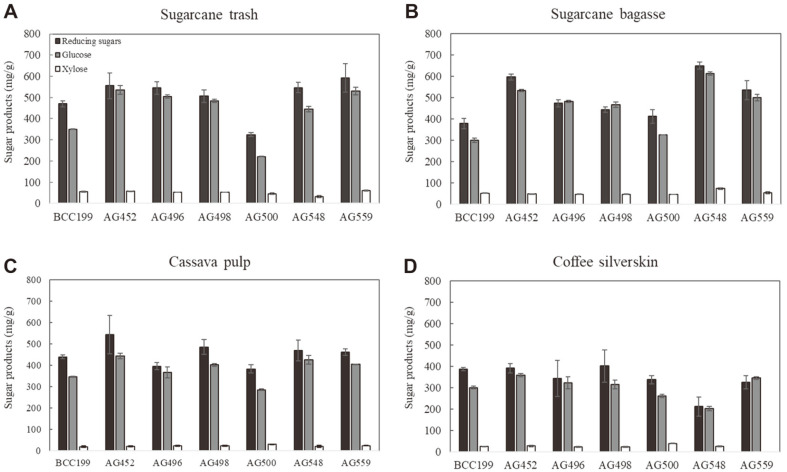
Enzymatic hydrolysis of pretreated sugarcane trash and bagasse, cassava pulp and coffee silverskin using secreted enzymes from newly isolated fungal strains.

Then, the potential of the crude enzymes produced by six candidate fungal strains for hydrolysis of the pretreated lignocellulosic biomasses was investigated. As shown in Fig. 4, the enzymes produced by the newly isolated strains were more efficient at hydrolyzing lignocellulosic biomass than BCC199 (the reference strain). Our previous research demonstrated that *A. aculeatus* BCC199 can produce a multienzyme complex that had high efficiency in the saccharification of alkaline-pretreated rice straw, corn cob, and corn stover [13]. However, the sugarcane bagasse and trash and cassava pulp tested in this study might not be compatible with BCC199 enzymes. This evidence supports the idea that no single-enzyme cocktail will be suitable for all applications [19]. Regarding the glucose yield, the *P. oxalicum* AG452 enzyme showed the highest glucose production of 532.98, 442.87, and 358.10 mg/g dried substrate (DS), corresponding to approximately 68.17%, 78.86%, and 66.84% cellulose conversion yield when pretreated sugarcane trash, cassava pulp or coffee silverskin were applied as substrates, respectively. In contrast to sugarcane bagasse, the use of the *Ta. siamensis* AG548 enzyme provided the highest glucose yield of 612.20 mg/g DS, corresponding to a 74.18% cellulose conversion yield. This evidence reflects the heterogeneity of lignocellulosic substrates, contributing to the optimum enzyme composition. In addition, Visser *et al*. [59] reported 63% glucan conversion obtained from hydrolysis of 8% (w/v) NaOH-pretreated bagasse using 20 FPU/g enzyme loading from a coculture of *Chrysoporthe cubensis* and *P. pinophilum*. Their conversion yield was less than that of our results when using pretreated bagasse hydrolysis.

Furthermore, after using the pretreated sugarcane trash and bagasse as substrates for hydrolysis, it was found that the reducing sugar and glucose yields were higher than those of pretreated cassava pulp and coffee silverskin. This might be due to the high lignin contents of pretreated cassava pulp and coffee silverskin, causing inhibition of the enzymatic hydrolysis of cellulose [60]. Other pretreatment methods should be applied for further improvement of the enzymatic hydrolysis of such substrates.

## Conclusion

The screening procedure using a miniaturized culture method for screening and selection of naturally isolated fungal strains for cellulase production was demonstrated in this study. The confirming results between a primary screening in 24-well plate culture and a secondary screening in 250-ml shake flasks represent a powerful procedure for our screening strategy. This method can be further applied to large isolation and screening programs of filamentous fungi when aiming to obtain a desired enzyme activity. In addition, this screening method offers direct screening in liquid culture, which is similar to the upscaled industrial production of certain enzymes. Furthermore, the second aim of this study was to find an enzyme suitable for the degradation of agro-industrial wastes, including sugarcane bagasse and trash, cassava pulp and coffee silverskin, the production of which is currently increasing. From the experiment, the *P. oxalicum* AG452 enzyme was the best fungal strain for saccharification of sugarcane trash, cassava pulp and coffee silverskin. Additionally, *Ta. siamensis* AG548 was regarded as the best strain for hydrolysis of sugarcane bagasse under our experimental conditions. Fungal strain improvement, *i.e.*, mutagenesis, genetic engineering techniques and bioprocess optimization, are needed to enhance the potential of these strains as onsite cellulase producers.

## Supplemental Materials

Supplementary data for this paper are available on-line only at http://jmb.or.kr.

## Figures and Tables

**Table 1:** Fungal species and isolation information of nine potential fungal taxa based on their cellulolytic enzyme production.

Fungal strain	Fungal species	Substrate origin	Collection site cl	Biosafety assification level^1^
AG438	*Aspergillus terreus*	Organic fertilizer	Tha Makham Natural Farming	BSL 2
			Center, Kanchanaburi Province	
AG452	*Penicillium oxalicum*	Dried butterfly pea	Tha Makham Natural Farming	BSL 1
			Center, Kanchanaburi Province	
AG466	*A. terreus*	Vermicompost	Tha Makham Natural Farming	BSL 2
			Center, Kanchanaburi Province	
AG496	*P. oxalicum*	Dried butterfly pea	Tha Makham Natural Farming	BSL 1
			Center, Kanchanaburi Province	
AG498	*P. oxalicum*	Dried butterfly pea	Tha Makham Natural Farming	BSL 1
			Center, Kanchanaburi Province	
AG499	*A. terreus*	Organic fertilizer	Tha Makham Natural Farming	BSL 2
			Center, Kanchanaburi Province	
AG500	*Trichoderma afroharzianum*	Soil	Tha Makham Natural Farming	BSL 1
			Center, Kanchanaburi Province	
AG548	*Talaromyces siamensis*	Soil	Tha Makham Natural Farming	BSL 1
			Center, Kanchanaburi Province	
AG559	*P. oxalicum*	Rice husk ash	Tha Makham Natural Farming	BSL 1
			Center, Kanchanaburi Province	

^1^BSL= Biosafety level based on the Thailand Biosafety Guidelines for Modern Biotechnology and Fungal Risk Assessment Report (http://www.biotec.or.th/biosafety/; [47].

**Table 2:** Activity of cellulase and hemicellulase and protein content based on selected fungal strains after cultivation in the production medium.

Strain number	Enzyme activity					Protein (mg/ml)

	Fpase (IU/ml)	CMCase (U/ml)	β-Glucosidase (U/ml)	Xylanase (U/ml)	β-Xylosidase (U/ml)	
BCC199	0.24 ± 0.01	13.91 ± 0.03	1.30 ± 0.00	64.88 ± 1.54	0.29 ± 0.02	0.45 ± 0.04
AG452	0.48 ± 0.02	13.67 ± 1.60	6.05 ± 0.27	37.88 ± 3.55	1.33 ± 0.11	0.44 ± 0.03
AG496	0.53 ± 0.04	20.16 ± 1.01	6.03 ± 0.23	94.55 ± 4.74	1.56 ± 0.07	0.53 ± 0.05
AG498	0.56 ± 0.03	14.93 ± 3.08	7.93 ± 0.25	90.35 ± 6.59	1.90 ± 0.04	0.55 ± 0.03
AG500	0.44 ± 0.04	15.32 ± 0.05	0.28 ± 0.02	40.70 ± 0.12	0.25 ± 0.03	0.63 ± 0.03
AG548	0.50 ± 0.01	14.90 ± 0.40	4.10 ± 0.27	43.38 ± 2.39	1.10 ± 0.04	0.48 ± 0.05
AG559	0.49 ± 0.03	15.92 ± 1.41	4.60 ± 0.49	45.79 ± 5.47	1.23 ± 0.12	0.43 ± 0.03

**Table 3:** Chemical composition of untreated and pretreated lignocellulosic biomasses.

Biomass	Untreated			Pretreated		

	Cellulose (%)	Hemicelluloses (%)	Lignin (%)	Cellulose (%)	Hemicelluloses (%)	Lignin (%)
Sugarcane bagasse	35.04	29.37	18.34	74.28	13.03	4.59
Sugarcane trash	28.95	25.50	17.60	70.61	15.24	4.90
Cassava pulp	20.18	13.21	18.31	50.55	9.84	19.09
Coffee silverskin	18.89	13.75	23.18	48.22	15.23	20.16
