# Supplementary material for: Towards a Miniaturized Culture Screening for Cellulolytic Fungi and Their Agricultural Lignocellulosic Degradation
Source: J Microbiol Biotechnol. 2020 Aug 27;30(11):1670–9. doi: 10.4014/jmb.2007.07005 (PMC9728337; doi:10.4014/jmb.2007.07005)
Supplement: Supplementary file 1 [file JMB-30-11-1670-supple.pdf]

J. Microbiol. Biotechnol.
https://doi.org/10.4014/jmb.2007.07005

**Table S1** GenBank accession number of the nine potential fungal taxa based on their cellulolytic enzyme production.

| **Fungal species** | **Isolate** | **GenBank Accession No.**[1] | | | | |
|---|---|---|---|---|---|---|
| | | **ITS** | ***TUB*** | ***CaM*** | ***RPB2*** | ***TEF-1α*** |
| *Aspergillus terreus* | AG438 | KY321322 | KY321331 | KY321322 | N/A | N/A |
| *Penicillium oxalicum* | AG452 | KY321323 | KY321322 | KY321322 | N/A | N/A |
| *Aspergillus terreus* | AG466 | KY321324 | KY321322 | KY321322 | N/A | N/A |
| *Aspergillus oxalicum* | AG496 | KY321325 | KY321322 | KY321322 | N/A | N/A |
| *Penicillium oxalicum* | AG498 | KY321326 | N/A | KY321322 | N/A | N/A |
| *Aspergillus terreus* | AG499 | KY321327 | KY321322 | KY321322 | N/A | N/A |
| *Trichoderma afroharzianum* | AG500 | KY321328 | KY321322 | KY321322 | KY321322 | KY321322 |
| *Talaromyces verruculosus* | AG548 | KY321329 | KY321322 | KY321322 | N/A | N/A |
| *Penicillium oxalicum* | AG559 | KY321330 | KY321322 | KY321322 | N/A | N/A |

[1] ITS = internal transcribed spacer; TUB = beta tubulin; CaM = calmodulin; RPB2 = second largest subunit of RNA polymerase II; TEF-1α = translation elongation factor 1α; N/A = information is not provided

**Table S2** Characteristics of lignocellulosic biomasses before and after pretreatment.

| Lignocellulosic biomass | Native | Pretreated |
| --- | --- | --- |
| Sugarcane trash | 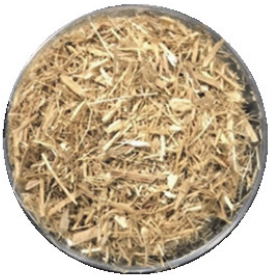 | 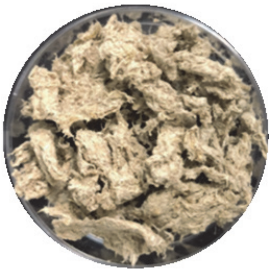 |
| Sugarcane bagasse | 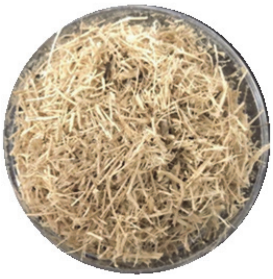 | 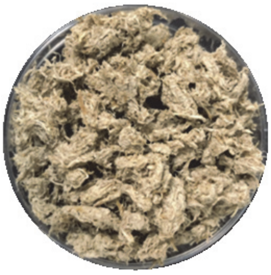 |
| Cassava pulp | 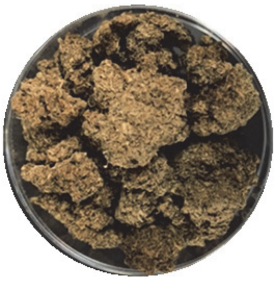 | 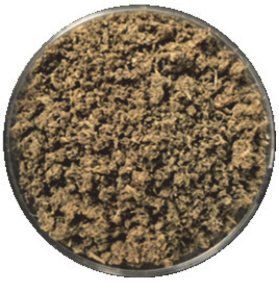 |
| Coffee silver skin | 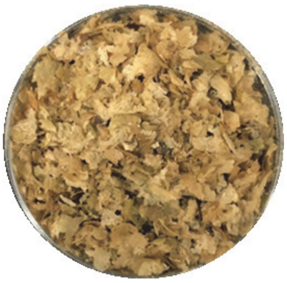 | 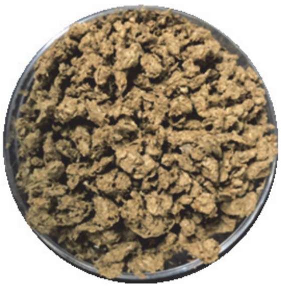 |

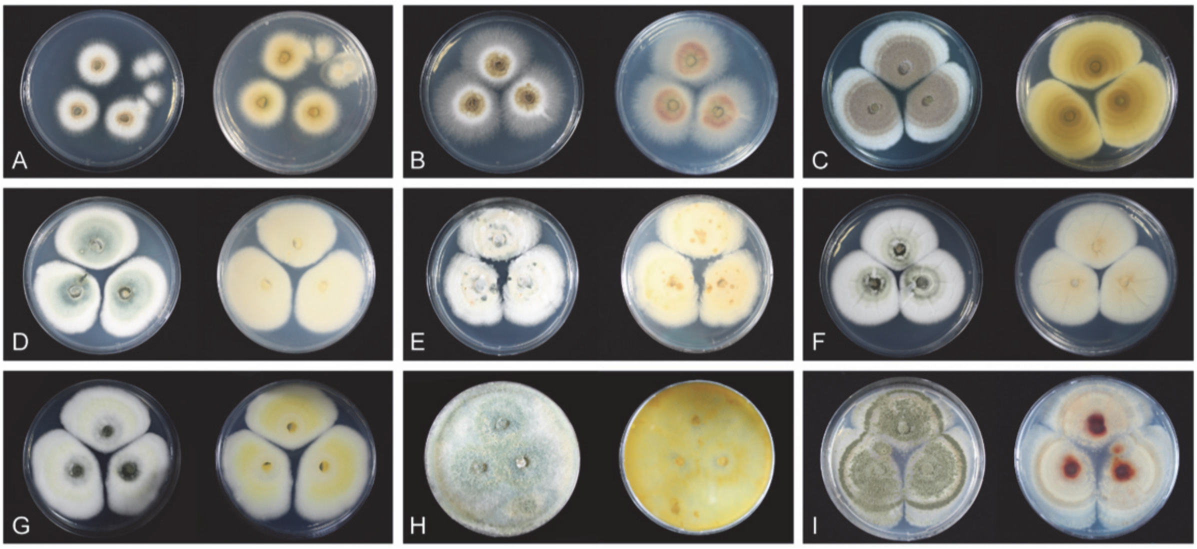


**Figure S1** Colonies from left (obverse) to right (reverse) on PDA after a 1-week incubation at room temperature: (A–C) *Aspergillus terreus*: (A) AG466, (B) AG438, and (C) AG499 (D–G) *Penicillium oxalicum*: (D) AG452, (E) AG496, (F) AG498, and (G) AG559 (H) *Trichoderma afroharzianum* (AG500) (I) *Talaromyces siamensis* (AG548).
